# Cytokine Secretion of Peripheral Blood Mononuclear Cells by* Hydnocarpus anthelminthicus* Seeds

**DOI:** 10.1155/2018/6854835

**Published:** 2018-06-03

**Authors:** Surang Leelawat, Kawin Leelawat

**Affiliations:** ^1^Faculty of Pharmacy, Rangsit University, Amphoe Mueang, Pathum Thani 12000, Thailand; ^2^Department of Surgery, Rajavithi Hospital, Bangkok 10400, Thailand

## Abstract

**Background:**

* Hydnocarpus anthelminthicus *is primarily used as a traditional medicine for the treatment of leprosy. Previous studies demonstrated that the clinical course of leprosy and the susceptibility to mycobacteria are recognized by the immune response of the host. The current study aims to investigate the effect of* H. anthelminthicus* seed oil and extracts on the secretion of cytokines from PBMCs involved in immune regulation.

**Methods:**

PBMCs from healthy volunteers were cultured and treated with LPS and* H. anthelminthicus* seed oil or extracts. Cell viability was detected with WST-1 cell proliferation assay reagent. Proinflammatory cytokines were quantified using ELISA with a specific antibody.

**Results:**

LPS-treated PBMCs significantly increased IL6 and TNF-*α* secretion.* H. anthelminthicus* seed oil had a synergistic effect with LPS on TNF-*α* secretion. The aqueous extract of* H. anthelminthicus* seed kernels and hulls significantly induced IL6 and TNF-*α* secretion. However, the ethanol extract of* H. anthelminthicus* seed kernels and hulls significantly decreased IL6, IL8, and TNF-*α* secretion in LPS-treated PBMCs.

**Conclusions:**

Extracts of* H. anthelminthicus* seeds demonstrated various effects on the proinflammatory cytokine secretion of PBMCs. The application of these extracts should depend on the immune response of the host, which determines the manifestation of the disease.

## 1. Introduction

*Hydnocarpus anthelminthicus* Pierre ex Laness. is a native Southeast Asian tree belonging to Flacourtiaceae.* Hydnocarpus* oil or chaulmoogra oil is prepared from the seeds of* Hydnocarpus* spp. and is primarily used as a traditional medicine for the treatment of leprosy [1].

Leprosy is a chronic disease caused by infection with* Mycobacterium leprae*. After infection, the immune response of the host determines the manifestation of the disease. Leprosy causes a peripheral neuropathy with potentially long-term disabilities [2, 3]. Its clinical spectrum ranges from tuberculoid leprosy through borderline forms to lepromatous leprosy of the Ridley-Jopling classification [4]. Tuberculoid leprosy is found in patients who have a strong cell-mediated immune response to* M. leprae*, limiting the disease to a few well-defined skin lesions [5]. Lepromatous leprosy is the severe form of leprosy presented with multiple skin involvements, neuropathy, blindness, and permanent disability. Patients with lepromatous leprosy have a poor cellular immune response but have high titers of ineffective antibodies against* M. leprae* [6].

However, there is no study of the effects of* H. anthelminthicus* on the immune response. In this study, the secretion of cytokines that are associated with immune responses for many systemic complications of infections, including IL6, IL8, IL10, and TNF-*α*, is measured from peripheral blood mononuclear cells (PBMCs) treated with* H. anthelminthicus*.

A recent study demonstrated that TNF-*α* induces the immune response toward the elimination of the pathogen and/or mediates the pathologic manifestations of the disease. TNF-*α* was induced following stimulation of PBMCs with total or components of* M. leprae*, namely, lipoarabinomannan [7]. A previous study found that lipoarabinomannan enhances LPS-induced TNF-*α* production by engaging scavenger receptors of PBMCs [8].

To stimulate the secretion of TNF-*α* and other proinflammatory cytokines, PBMCs were treated with LPS. In addition, the modulation effect of* H. anthelminthicus *on cytokine secretion in LPS-treated PBMCs was examined.

## 2. Materials And Methods

### 2.1. Sample Preparation

*Hydnocarpus anthelminthicus* from Lampang province, Thailand, was cleaned, dried, and then separated into seed hulls and kernels. Seed kernels were cleaned, dried at 50°C for 30 min, ground into paste, and pressed using a hydraulic presser. Seed kernel marc was then dried at 50°C for 30 min, ground into paste, and extracted with 80% ethanol. Marc from the ethanol extract was then extracted with water by heating in a water bath for 30 min, filtered, and evaporated using a freeze dryer.* H. anthelminthicus* seed oil (HSO), ethanol extract of* H. anthelminthicus* seed kernels (EHK), and aqueous extract of* H. anthelminthicus* seed kernels (AHK) were stored at -80°C for further study.

*H. anthelminthicus* hulls were dried at 50°C for 30 min, ground into powder, and extracted with 80% ethanol. Marc from the ethanol extract was then extracted with water by heating on a water bath for 30 min, filtered, and evaporated using a freeze dryer. Ethanol extract of* H. anthelminthicus* seed hulls (EHH) and aqueous extract of* H. anthelminthicus* seed hulls (AHH) were stored at -80°C for further study.

### 2.2. Isolation and Culture of Peripheral Blood Mononuclear Cells

Blood samples were obtained from healthy volunteers at Rajavithi Hospital after approval by the Rajavithi Ethics Committee. Six healthy volunteers (three males and three females) aged 20-40 years with no diseases involved in immune response including acquired immune deficiency syndrome (AIDS), diabetes mellitus, and autoimmune diseases participated in the study. Peripheral blood mononuclear cells (PBMCs) were separated from 25 ml blood using Ficoll-Paque density centrifugation (Pharmacia, Piscataway, NJ, USA). PBMCs were washed with phosphate-buffered saline (PBS) and then cultured in Ham's F12 (Gibco, Grand Island, NY, USA) treated with 10% heat-inactivated fetal calf serum (Gibco), 100 U/ml penicillin, and 100 *μ*g/ml streptomycin (Gibco) at 37°C in a 5% CO_2_ humidified atmosphere.

### 2.3. The Effect of* H. Anthelminthicus* Seed Oil and Extracts on PBMC Proliferation

PBMCs were seeded in 96-well tissue culture plates at a density of 10,000 cells per well in Ham's F12 at 37°C in a 5% CO_2_ humidified atmosphere for 24 hours. Lipopolysaccharide (LPS; 10 *μ*g/ml) was added to PBMC culture with HSO at concentrations of 0, 5, 10, and 20 *μ*l/ml or* H. anthelminthicus *extracts (EHH, EHK, AHH, or AHK) at concentrations of 0, 5, 10, and 20 *μ*g/ml. The cells were subsequently incubated for 24 hours before applying WST-1 cell proliferation assay reagent (Roche Diagnostics, Laval, Quebec) according to the manufacturer's instructions. The percentage of cell proliferation was calculated relative to control PBMCs. The proliferation of control PBMCs at 24 hours was indicated as 100%.

### 2.4. The Effect of* H. Anthelminthicus* Seed Oil and Extracts on Proinflammatory Cytokine Secretion

PBMCs (5 × 10^5^ cells/ml) in 12-well tissue culture plates were cultured in Ham's F12 at 37°C in a 5% CO_2_ humidified atmosphere for 24 hours. Lipopolysaccharide (LPS; 10 *μ*g/ml) was added to the PBMC culture with HSO at concentrations of 0, 5, 10, and 20 *μ*l/ml or* H. anthelminthicus* extracts (EHH, EHK, AHH, and AHK) at concentrations of 0, 5, 10, and 20 *μ*g/ml. PBMCs were subsequently incubated for 24 hours. The supernatant was collected for quantitative analysis of proinflammatory cytokines (IL6, IL8, IL10, and TNF-*α*) using an enzyme-linked immunosorbent assay kit with a specific antibody to each cytokine (Quantikine® Colorimetric Sandwich ELISA, R&D Systems, Minneapolis, MN) according to the manufacturer's instructions. Briefly, supernatants from control and treated samples were added to each well of a 96-well plate and incubated for 2 hours at room temperature. After washing, human IL6 (IL8, IL10, or TNF-*α*) conjugate (horseradish peroxidase) was added and incubated for 2 hours at room temperature. Then, the samples were washed and incubated with substrate solution [stabilized hydrogen peroxide and stabilized chromogen (tetramethylbenzidine)] for 20 min at room temperature and protected from light. A 50 *μ*l stop solution (2N sulfuric acid) was added to each well. The color in the wells changed from blue to yellow. The optical density of each well was determined within 30 minutes, using a microplate reader set to 450 nm.

### 2.5. Statistical Analysis

The experiments were performed in triplicate, and quantitative data were described as the mean ± standard deviation. Data between three or more groups were compared using one-way analysis of variance (ANOVA) followed by Dunnett's post hoc test. A* p* value of less than 0.05 was considered to indicate a statistically significant result.

## 3. Results

### 3.1. The Effect of* H. Anthelminthicus* Seed Oil and Extract on PBMC Proliferation

**Figure 1:**
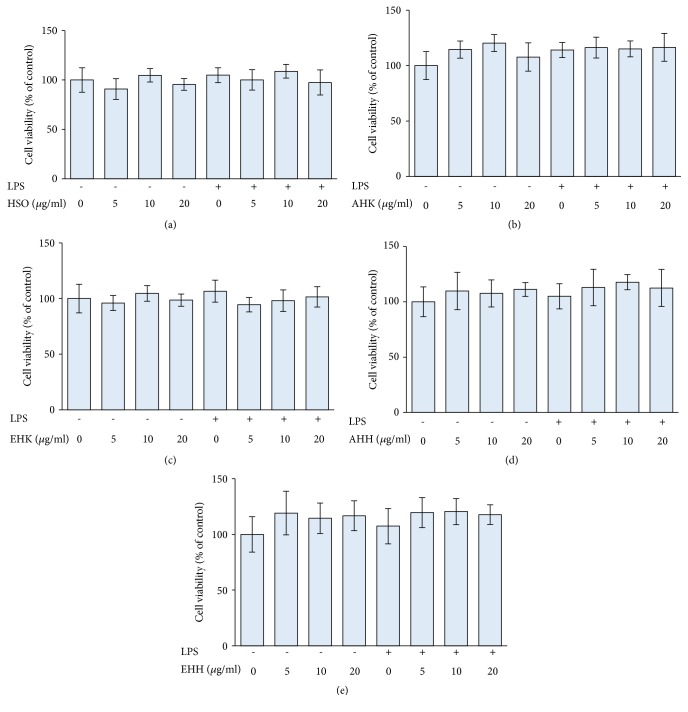
The effect of HSO (a), AHK (b), EHK (c), AHH (d), and EHH (e) on PBMC proliferation. PBMCs were separated using Ficoll-Paque density centrifugation, seeded in 96-well plates and treated with 10 *μ*g/ml LPS and extracts at the indicated concentrations. The cells were then incubated for 24 h before the addition of WST-1 cell proliferation assay reagent.

PBMCs and LPS-treated PBMCs were cultured with 5-20 *μ*g/ml HSO, AHK, EHK, AHH, or EHH. The results demonstrated that LPS and all extracts of* H. anthelminthicus* seeds had no effect on PBMC proliferation (Figure 1).

### 3.2. The Effect of* H. Anthelminthicus* Seed Oil and Extracts on Proinflammatory Cytokine Secretion

#### 3.2.1. The Effect of HSO on Cytokine Secretion

**Figure 2:**
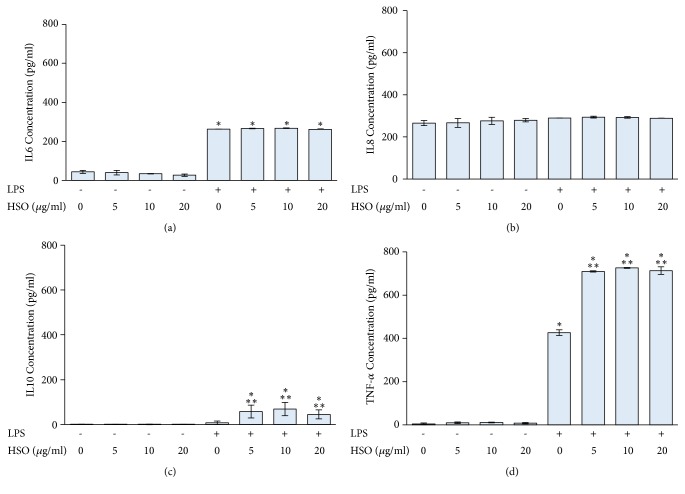
The effect of HSO on IL6 (a), IL8 (b), IL10 (c), and TNF-*α* (d) production in PBMCs and LPS-treated PBMCs. PBMCs were separated using Ficoll-Paque density centrifugation, seeded in 12-well plates, and treated with 10 *μ*g/ml LPS and HSO at the indicated concentration for 24 h. Levels of IL6, IL8, IL10, and TNF-*α* secretion were determined using ELISA with specific antibodies to each cytokine. *∗ p* < 0.05 compared to control group; *∗∗ p* < 0.05 compared to LPS-treated group alone.

PBMCs were cultured with 5-20 *μ*g/ml HSO alone or 5-20 *μ*g/ml HSO with 10 *μ*g/mg LPS. The results demonstrated that LPS induced IL6 and TNF-*α* secretion of PBMCs but had no effect on IL8 and IL10 secretion. HSO significantly induced TNF-*α* and IL10 secretion in LPS-treated PBMCs at concentrations of 5-20 *μ*g/ml. However, HSO had no effect on the secretion of IL6 and IL8 in PBMCs and LPS-treated PBMCs (Figure 2).

#### 3.2.2. The Effect of AHK on Cytokine Secretion

**Figure 3:**
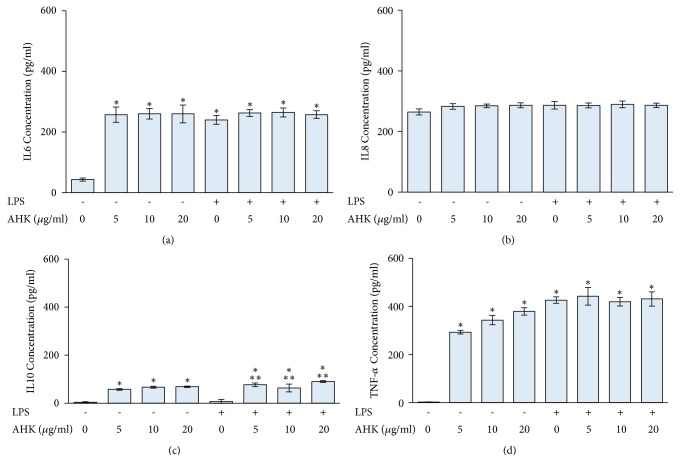
The effect of AHK on IL6 (a), IL8 (b), IL10 (c), and TNF-*α* (d) production in PBMCs and LPS-treated PBMCs. PBMCs were separated using Ficoll-Paque density centrifugation, seeded in 12-well plates, and treated with 10 *μ*g/ml LPS and AHK at the indicated concentrations for 24 h. The levels of IL6, IL8, IL10, and TNF-*α* secretion were determined using ELISA with specific antibodies to each cytokine. *∗ p* < 0.05 compared to control group; *∗∗ p* < 0.05 compared to LPS-treated group alone.

LPS-treated PBMCs showed significantly increased IL6 and TNF-*α* secretion, although the secretion of IL8 and IL10 was not different. Treatment with AHK significantly induced IL6, IL10, and TNF-*α* secretion in PBMCs. AHK and LPS treatment significantly induced IL10 secretion but had no effect on IL6, IL8, and TNF-*α* secretion (Figure 3).

#### 3.2.3. The Effect of EHK on Cytokine Secretion

**Figure 4:**
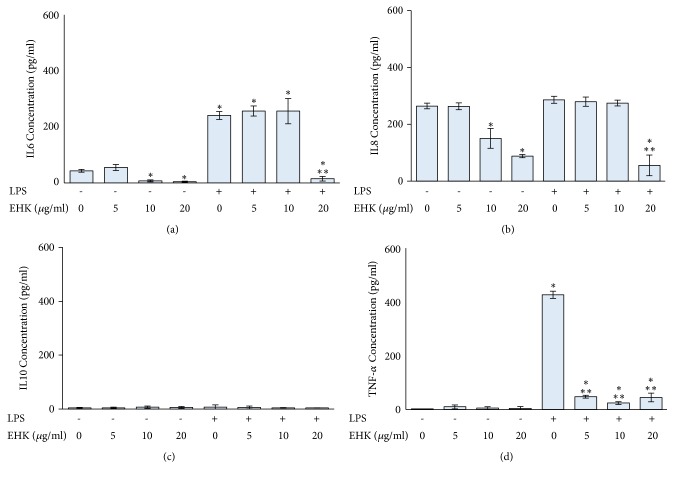
The effect of EHK on IL6 (a), IL8 (b), IL10 (c), and TNF-*α* (d) production in PBMCs and LPS-treated PBMCs. PBMCs were separated using Ficoll-Paque density centrifugation, seeded in 12-well plates, and treated with 10 *μ*g/ml LPS and EHK at the indicated concentrations for 24 h. The levels of IL6, IL8, IL10, and TNF-*α* secretion were determined using ELISA with specific antibodies to each cytokine. *∗ p* < 0.05 compared to control group; *∗∗ p* < 0.05 compared to LPS-treated group alone.

LPS treatment alone significantly induced IL6 and TNF-*α* secretion, while the secretion of IL8 and IL10 was not different from control PBMCs. Treatment with EHK significantly decreased the secretion of IL6 and IL8 at concentrations of 10 and 20 *μ*g/ml. However, EHK had no effect on IL10 and TNF-*α* secretion. EHK and LPS treatment significantly decreased the secretion of IL6 and IL8 at a concentration of 20 *μ*g/ml and the secretion of TNF-*α* at a concentration of 5-20 *μ*g/ml. However, EHK had no effect on IL10 secretion in PBMCs and LPS-treated PBMCs (Figure 4).

#### 3.2.4. The Effect of AHH on Cytokine Secretion

**Figure 5:**
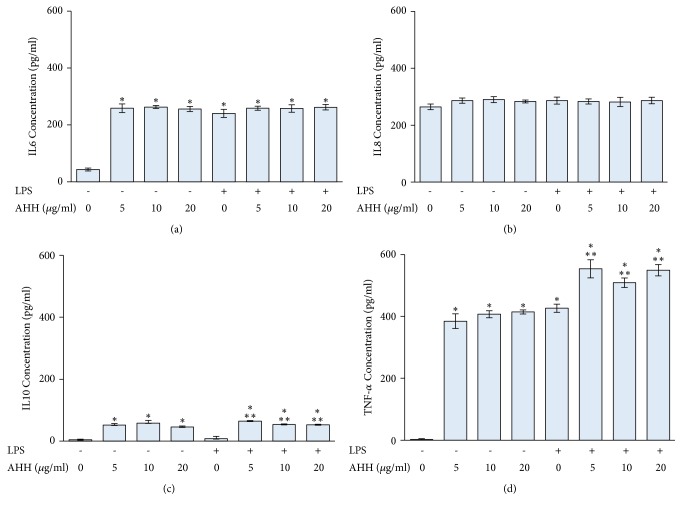
The effect of AHH on IL6 (a), IL8 (b), IL10 (c), and TNF-*α* (d) production in PBMCs and LPS-treated PBMCs. PBMCs were separated using Ficoll-Paque density centrifugation, seeded in 12-well plates, and treated with 10 *μ*g/ml LPS and AHH at the indicated concentrations for 24 h. The levels of IL6, IL8, IL10, and TNF-*α* secretion were determined using ELISA with specific antibodies to each cytokine. *∗ p* < 0.05 compared to control group; *∗∗ p* < 0.05 compared to LPS-treated group alone.

Treatment with LPS significantly induced the levels of IL6 and TNF-*α* in PBMCs, whereas IL8 and IL10 levels were not altered. AHH treatment significantly increased IL6, IL10, and TNF-*α* secretion in PBMCs but had no effect on IL8 secretion. Additionally, treatment with AHH and LPS significantly induced IL10 and TNF-*α* secretion of PBMCs but had no effect on IL6 and IL8 (Figure 5).

#### 3.2.5. The Effect of EHH on Cytokine Secretion

**Figure 6:**
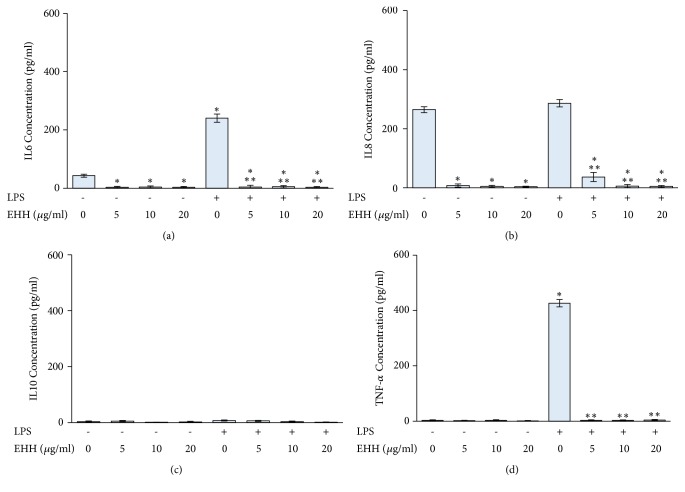
The effect of EHH on IL6 (a), IL8 (b), IL10 (c), and TNF-*α* (d) production in PBMCs and LPS-treated PBMCs. PBMCs were separated using Ficoll-Paque density centrifugation, seeded in 12-well plates, and treated with 10 *μ*g/ml LPS and EHH at the indicated concentration for 24 h. The levels of IL6, IL8, IL10, and TNF-*α* secretion were determined using ELISA with specific antibodies to each cytokine. *∗ p* < 0.05 compared to control group; *∗∗ p* < 0.05 compared to LPS-treated group alone.

LPS treatment alone significantly released IL6 and TNF-*α* in PBMCs. However, LPS had no influence on IL8 and IL10 secretion. EHH treatment significantly decreased the secretion of IL6 and IL8 at concentrations of 5, 10, and 20 *μ*g/ml in PBMCs. However, EHH had no effect on IL10 and TNF-*α* secretion. In LPS-treated PBMCs, EHH significantly decreased the secretion of IL6, IL8, and TNF-*α* at concentrations of 5, 10, and 20 *μ*g/ml. However, EHH did not influence the level of IL10 in LPS-treated PBMCs (Figure 6).

## 4. Discussion

The pathogenesis and clinical manifestation of leprosy are determined by the polarization of the immune response specific to* M. leprae* [9]. Consequently, the effect of drugs on cytokine secretion associated with the immunological response should play an essential role in leprosy treatment.

Presently, the components of* H. anthelminthicus* seed oil have not been reported. However, the composition of certain chaulmoogra oil has been studied. Chaulmoogra oil from* H. kurzii* is composed of triglycerides [70-80% glycerides of cyclopentenyl fatty acid (hydnocarpic, chaulmoogric, and gorlic acids)] and palmitic, stearic, and oleic acids [10]. Chaulmoogra oil from* H. wightiana* is composed of triglycerides (80-90% glycerides of cyclopentenyl fatty acid) and palmitic, oleic, and steric acids [10]. Chaulmoogra oil from* H. wightiana* contains 48% hydnocarpic acid, 27% chaulmoogric acid, gorlic acid, and other acids [11]. Three flavonolignans (hydnowightin, hydnocarpin, and neohydnocarpin) isolated from* H. wightiana* seeds established potent hypolipidemic activity in mice [12]. A new phenolic glycoside isolated from the bark of* H. annamensis* exhibited COX-2 inhibitory activity in vitro [13]. Hydnocarpin and isohydnocarpin from the seed hulls of* H. wightiana* exhibited free radical scavenging, *α*-glucosidase, and moderate N-acetyl-*β*-D glucosaminidase inhibitory activities, which may be responsible for antidiabetic properties [14].

In this study, LPS induced proinflammatory cytokine (IL6 and TNF-*α*) secretion of PBMCs but had no effect on chemoattractant cytokine IL8 and anti-inflammatory cytokine IL10. HSO had no effect on the proliferation and cytokine secretion of PBMCs. However, HSO showed synergism with LPS on TNF-*α* secretion of PBMCs. Consequently, the use of HSO might be imprudent for patients infected with Gram-negative bacteria due to the induction of proinflammatory cytokines.

Our study demonstrated that EHH and EHK inhibit the release of inflammatory cytokines from PBMCs and LPS-treated PBMCs. A previous study demonstrated that hydnocarpin, the main product among flavonolignans, showed good anti-inflammatory and antineoplastic activity in vivo [12]. The major cyclopentenyl fatty acids, including gorlic acid, chaulmoogric acid, and hydnocarpic acid, from seeds of* Carpotroche brasiliensis* demonstrated significant oral anti-inflammatory and peripheral antinociceptive effects in vivo [15]. Furthermore, silybin, the major flavonolignan from* Silybum marianum* seed extracts, demonstrated an inhibitory effect on LPS-induced production of TNF-*α* and IL-1*β* and activation of NF-*κ*B and the NLRP3 inflammasome in mice [16]. It is suggested that EHH and EHK might contain flavonolignan-like compounds that inhibit the proinflammatory cytokine secretion of PBMCs. Flavonolignan is soluble in organic solvent. The aqueous extract of* H. anthelminthicus* seed should not contain flavonolignan. However, AHH and AHK induce proinflammatory cytokine (IL6 and TNF-*α*) production in PBMCs similar to LPS stimulation. It is implicit that AHH and AHK might induce inflammation. Consequently, the usage of* H. anthelminthicus* seed extract should be consciously considered.

This is the first study of* H. anthelminthicus* for the immune response. Our study explored the effect of* H. anthelminthicus* seed kernel and hull extracts on proinflammatory cytokine (IL6, IL8, and TNF-*α*) production. However, the chemical constituents of* H. anthelminthicus* seed hulls and kernels are unknown. Fractions of* H. anthelminthicus* demonstrated various effects. Consequently, the solvent and extraction process should be verified for the therapeutic application of* H. anthelminthicus*.

AHH and AHK induced proinflammatory cytokine. We suggested that AHH and AHK will be a great benefit for patients with lepromatous leprosy who have a poor cellular immune response against* M. leprae*. Meanwhile, the ethanol extracts of* H. anthelminthicus* seed hulls and kernels demonstrated an inhibitory effect on proinflammatory cytokine secretion. They might influence the downregulation of innate immune and cell-mediated immune responses due to the inhibition of IL6, IL8, and TNF. These extracts should be used depending on the immune response of the host, which determines the manifestation of the disease. Consequently, the inhibitory effect on proinflammatory cytokines should be considered as anti-inflammatory agents for the treatment of inflammatory diseases, including systematic inflammatory response syndrome (SIRS), severe sepsis, cancers, and autoimmune diseases.

Further studies should be performed to elucidate the chemical constituents of* H. anthelminthicus* seed hulls and kernels. Because of the study design limitations, this research was conducted with PBMCs from healthy volunteers. Consequently, further study should be performed using PBMCs from leprosy patients or PBMCs treated with* M. leprae *antigen to study the effect of* H. anthelminthicus *on cytokines secretion. Moreover, an* in vivo* study is important to investigate the potential application of* H. anthelminthicus* for the clinical treatment of inflammation in a variety of diseases caused by a severe immune response.

## Data Availability

The data used to support the findings of this study are available from the corresponding author upon request.
